# Spatial and Temporal Profiling of Griseofulvin Production in *Xylaria cubensis* Using Mass Spectrometry Mapping

**DOI:** 10.3389/fmicb.2016.00544

**Published:** 2016-04-26

**Authors:** Vincent P. Sica, Evan R. Rees, Edem Tchegnon, Robert H. Bardsley, Huzefa A. Raja, Nicholas H. Oberlies

**Affiliations:** Department of Chemistry and Biochemistry, University of North Carolina at Greensboro, GreensboroNC, USA

**Keywords:** griseofulvin, polyhydroxyanthraquinones, fungal secondary metabolites, co-culture, endophytes, mass spectrometry mapping

## Abstract

A large portion of natural products research revolves around the discovery of new, bioactive chemical entities; however, studies to probe the biological purpose of such secondary metabolites for the host organism are often limited. Mass spectrometry mapping of secondary metabolite biosynthesis *in situ* can be used to probe a series of ecological questions about fungi that may be lost through traditional natural products chemistry extraction protocols. A griseofulvin-producing fungal culture of the Xylariaceae family, isolated as an endophyte of the tree *Asimina triloba*, was analyzed through a series of spatial and temporal mapping experiments. This fungus produced unique fungal characteristics, such as guttates and stroma, both of which were explored spatially. The distribution of griseofulvin on this culture in isolation was compared to its dispersal when grown in co-culture with a competing *Penicillium* species via a droplet–based surface sampling system. The fungistatic properties of griseofulvin were visualized, including the consequences for biosynthesis of polyhydroxyanthraquinones in a rival culture.

## Introduction

**FIGURE 1:**
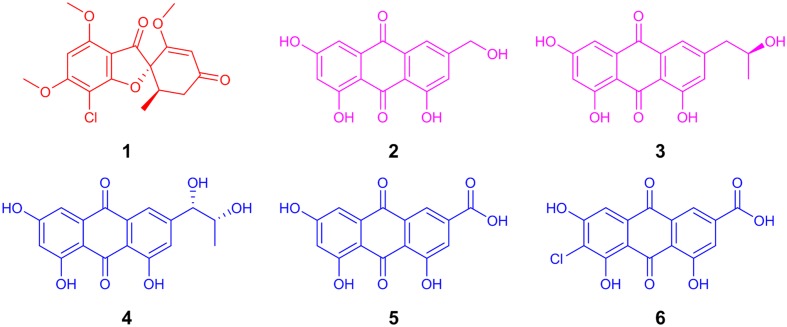
**The structure of the antifungal agent, griseofulvin **(1**; red), from *Xylaria cubensis* (G536).** The polyhydroxyanthraquinones **(2–6)** from *Penicillium restrictum* (G85) were grouped into two categories (purple and blue) based on their distributions in co-culture.

For decades, griseofulvin (**Figure 1**) has been well studied, imparting a rich history in mycology, structure elucidation, and biological activity (Petersen et al., 2014). For mycologists, its initial discovery from *Penicillium griseofulvum* (Oxford et al., 1939) and subsequently *P. janczewskii* (Brian, 1946; McGowan, 1946) proved noteworthy as these griseofulvin-producing cultures induced abnormal development of fungal hyphae. Essentially, griseofulvin triggered other fungal hyphae to “curl” imparting the name “curling factor” as its original descriptor (Brian, 1946; McGowan, 1946). From the perspective of organic chemistry, the characterization of griseofulvin details how the structure elucidation of fungal metabolites evolved in the 20th century. Initially, an ensemble of IR and UV spectroscopy, coupled with combustion analysis of derivatives or degradation products, was employed (Oxford et al., 1939; Grove and McGowan, 1947; Grove et al., 1952). Ultimately, ^1^H NMR (Levine and Hicks, 1971) and X-ray crystallography (Malmros et al., 1977) were used to support the previously proposed structures. From a biological activity standpoint, griseofulvin was originally noted to have a unique effect on molds (Grove and McGowan, 1947). Griseofulvin has been employed to treat fungal infections (Gentles, 1958; Williams et al., 1958), notably dermatophytosis (ringworm), and ultimately became a commercialized product in 1975 (e.g., Fulvicin, Gris-PEG, Grifulvin V). More recently, griseofulvin has shown potential by inhibiting the proliferation of cancer cells but with low general toxicity (Ho et al., 2001; Kim et al., 2011; Liéby-Muller et al., 2015).

As an antifungal agent, griseofulvin is fungistatic (Robinson, 1960; Corvis et al., 2006) as opposed to fungicidal. This denotes that it inhibits fungal growth, rather than kills competing fungi. While this inhibition has been observed biologically via agar-based disk diffusion assays (Nweze et al., 2010), the spatial and temporal distribution of its chemistry has never before been visualized. Advances in ambient mass spectrometry techniques, such as mass spectrometry imaging, have allowed for the mapping of secondary metabolites *in situ* (Hsu and Dorrestein, 2014; Jarmusch and Cooks, 2014). However, only a few recent studies have explored mass spectrometry mapping experiments of fungi *in situ* (Sica et al., 2014, 2015), and even fewer have attempted to understand the chemical interactions between competing microbial cultures (Moree et al., 2013).

The popular ambient ionization mass spectrometry techniques include matrix assisted laser desorption electrospray ionization (MALDI), desorption electrospray ionization (DESI), and nanoDESI to achieve surface sampling and mapping of metabolites directly on fungal cultures. While effective, each of these techniques is not without limitations. Comparatively, MALDI provides superior spatial resolution, but it often requires the application of a matrix to the sample, which can cause ion suppression when dealing with small molecules. Also, it destroys the sample during the laser desorption/ionization process, thus limiting repeat analysis of a growing fungal culture. DESI is less destructive, but the gas and solvent pressures used for this system can manipulate the surface of the fungal culture and its surrounding environment, making it difficult to analyze directly (Sica et al., 2014; Tata et al., 2015). NanoDESI overcomes some of these issues by forming a liquid microjunction with the surface of the culture. This technique is even less abrasive than DESI and more amenable for surface sampling of a fungus and its surrounding agar. However, certain characteristics, such as aerial fungal hyphae and guttates (liquid droplets; Figueroa et al., 2014), have been known to clog the system (Watrous et al., 2012). Furthermore, both DESI and nanoDESI are not amenable to the natural heterogeneous topography of a fungal culture (Sica et al., 2014, 2015).

To overcome these issues, a droplet–liquid microjunction–surface sampling probe (droplet–LMJ–SSP) (Kertesz and Van Berkel, 2010, 2013) was optimized to sample and map the secondary metabolites of fungal cultures *in situ* (Sica et al., 2015). This technique is robust and provides a non-destructive sampling system that is tolerant of the topography that fungal cultures possess. Additionally, it has great ionization efficiency and reliability due to its use of electrospray ionization (ESI). The incorporation of liquid chromatography is also beneficial, since it provides mutually supportive data, including retention time and UV data, when analyzing fungal metabolites. The summation of these data, and the ability to separate complex mixtures via chromatography, imparts a high degree of confidence when assigning structures.

Previously, griseofulvin was discovered from xylariaceous endophytes, an important taxonomic group of fungi (Park et al., 2005; Richardson et al., 2014). Endophytes of the *Xylaria* generate morphologically distinct stromatic outgrowths, but the spatial distribution of secondary metabolites throughout the stroma is poorly understood (Stadler et al., 2006; Bills et al., 2012; Pažoutová et al., 2013). Recently, fungal endophytes from the tree *Asimina triloba* were isolated and revealed several species of xylariaceous fungi, one of which biosynthesized griseofulvin. Mapping the chemical entities of this fungus as it interacts with a competing fungus *in situ* can begin to answer a series of ecological questions that may be lost through a traditional natural products extraction protocol: *where* are the metabolites distributed (spatial), *when* is each metabolite formed (temporal), *what* metabolites are interacting (qualitative), *how much* of each metabolite is produced (quantitative), *why* do the fungi produce them (function), and *which* fungus is most affected by the interaction (target). This project sought to probe these questions by using the droplet–LMJ–SSP for direct analysis of a griseofulvin-producing endophytic fungus.

## Materials and Methods

### Isolation of Fungal Cultures

Both fungal species employed in this study were isolated as endophytes from surface sterilized plant tissue segments. Fungal strain G536 was isolated from surface sterilized twigs of paw paw (*A. triloba* (L.) Dunal, Annonaceae) collected from Pfafftown, NC, USA (36°09′58.8″N 80°24′18.6″W). Fungal strain G85 was isolated from surface sterilized stems of a milk thistle [*Silybum marianum* (L.) Gaertn. Asteraceae] (Figueroa et al., 2014). Isolation of fungal endophytes was performed using methodology described previously (Vandermolen et al., 2013; Raja et al., 2015b). These fungal cultures are maintained at the University of North Carolina at Greensboro, Department of Chemistry and Biochemistry Fungal Culture Collection, under voucher numbers G536 and G85, respectively.

### Identification of Fungal Cultures

Both strains were identified via morphological and molecular methods. Since the former could only provide information regarding the genus of the fungal isolates, molecular data were used to obtain a more complete identification.

For strain G536, the partial ribosomal polymerase II subunit 2 (*RPB2*) gene was amplified using primers RPB2-5f and RPB2-7cR primers (Liu et al., 1999). DNA extraction, PCR amplification and Sanger sequencing was performed using protocols outlined previously (Malkus et al., 2006; Figueroa et al., 2014). Typically, we acquire genomic DNA from 2 week old cultures on PDA (El-Elimat et al., 2014; Figueroa et al., 2014); however, this proved challenging for culture G536 and each attempt to acquire DNA from 2 week old cultures proved unsuccessful. Interestingly, to overcome this challenge, DNA was acquired from a younger (1 week) culture grown on 10 ml of YESD. Methods utilized for the molecular identification of strain G85 have been outlined previously (Figueroa et al., 2014).

Two forward and reverse contigs of the partial *RPB2* regions were obtained for strain G536 and were assembled and edited using Sequencher v5.3 (Gene Codes Corporation, Ann Arbor, MI, USA). The consensus sequence was then submitted to NCBI GenBank database via BLAST search to obtain matches with identical sequences for subsequent phylogenetic analysis. The BLAST search revealed *Xylaria cubensis* (GQ848365, GQ848364, GQ848366, and GQ853017), as the top match with high coverage and percent identity values. Therefore, these sequences, along with other *RPB2* sequences of *Xylaria* obtained for a recent multi-gene phylogenetic evaluation of Xylariaceae (Hsieh et al., 2010), were downloaded and incorporated into a multiple alignment for maximum likelihood (ML) analysis with RAxML using previously described methods (Raja et al., 2015a).

In addition to the *RPB2* gene, the entire ITS region was PCR-amplified using primer combinations ITS5 and ITS4 (White et al., 1990; Gardes and Bruns, 1993) using PCR amplification protocols defined previously (Figueroa et al., 2014). A forward and reverse contig was obtained as above using Sequencher. The ITS sequence was then subjected to a BLAST search using NCBI GenBank database. Based on this, the closest hits were members of the genus *X. cubensis*, Ascomycota [*X. cubensis* (Mont.) Fr., GenBank GU991523; Identities = 386/392 (98%); Gaps = 0/392 (0%), *X. cubensis* GenBank AB625440; Identities = 383/392 (98%); Gaps = 0/392 (0%)]. In addition, our ITS sequence also showed high coverage and percent identity values with 26 unidentified sequences of Sordariomycetes, Ascomycota [GenBank JQ761856, JQ761796, JQ761749, JQ761744, JQ761693, JQ761562, JQ761423, JQ761405, JQ761381, JQ760963, JQ760763, JQ760481, JQ760128, JQ761454, JQ760138, JQ761695, JQ761549, JQ761384, JQ761371, JQ761326, JQ761317, JQ760708, JQ760193, JQ761847, JQ761823, JQ761727; Identities = 391/392 (99%); Gaps = 0/392 (0%)]. Interestingly, these ITS sequences were isolated as endophytes from lichen fungi (endolichenic) collected from Highlands Biological Station, North Carolina (U’Ren et al., 2012), which was within 200 miles from where fungal culture G536 was collected. The top BLAST matches were downloaded and incorporated into a multiple alignment for ML analysis with RAxML as above.

### Fermentation of Fungal Cultures

In preparation for chemical extraction, G536 was grown on rice, as this has been shown to be an efficient medium for the production of secondary metabolites in culture (Vandermolen et al., 2013). To make seed cultures for inoculating rice, a piece of fresh culture grown in potato dextrose (PD) (Difco) or 2% malt extract (ME) (Difco) media was excised from the leading edge of the colony and transferred to a liquid seed medium containing 2% soy peptone, 2% dextrose, and 1% yeast extract (YESD; 5 g of yeast extract, 10 g of soy peptone, and 10 g of D-glucose in 500 ml distilled water). Following incubation for 7 days at 22°C with agitation, the culture was used to inoculate 10 g of rice media prepared using 30 ml of distilled H_2_O and autoclaved in a 250 ml Erlenmeyer flask. This screener scale fermentation was incubated at 22°C for 14–21 days prior to chemical extraction. For large-scale production of fungal cultures, four 250 ml Erlenmeyer flasks containing 10 g of rice were inoculated using one seed culture for each flask.

### Extraction of Fungal Culture

The fungal culture, coded G536, was extracted using a previously reported procedure (Ayers et al., 2011; Figueroa et al., 2012; El-Elimat et al., 2014). Briefly, the fungus was extracted by adding 60 ml of MeOH–CHCl_3_ (1:1) to a 250 ml flask containing 10 g of rice with endophytic fungal growth. The fungus was chopped with a spatula before shaking overnight (∼16 h) at ∼100 rpm at room temperature. Using vacuum filtration, the sample was filtered, and the remaining residue was washed with MeOH. To the filtrate, 90 ml of CHCl_3_ and 150 ml of H_2_O were added. The mixture was stirred for 30 min and then transferred into a separatory funnel. The organic layer was drawn off and evaporated to dryness. The dried organic extract was re-constituted in 100 ml of MeOH–CH_3_CN (1:1) and 100 ml of hexanes and transferred to a separatory funnel. The biphasic solution was shaken vigorously. The MeOH–CH_3_CN layer was evaporated to dryness under vacuum (69 mg).

### Isolation and Identification of Griseofulvin

The extracted material (69 mg) was dissolved in CHCl_3_, adsorbed onto Celite 545, and fractionated via normal phase flash chromatography on a CombiFlash Rf system using a 4 g RediSep Rf Si-gel Gold column (Teledyne-Isco, Lincoln, NE, USA). The gradient solvent system was hexane–CHCl_3_–MeOH at a flow rate of 18 ml/min with 72.9 column volumes over 19.4 min. This afforded three fractions: fraction 1 (0.8 mg), fraction 2 (10 mg), and fraction 3 (50 mg). Fraction 2 was subjected to preparative HPLC using a gradient system of 40 to 100% CH_3_CN in H_2_O (acidified with 0.1% formic acid) over 30 min at a flow rate of 21.1 ml/min on a Kinetex C_18_ column (Phenomenex, Torrance, CA, USA; 5 μm; 250 mm × 21.2 mm). Griseofulvin eluted at 6.9 min and yielded 1.06 mg. The structure of griseofulvin was verified (**Supplementary Figure S1**; **Supplementary Table S1**) via NMR on a JEOL ECS-400 NMR spectrometer (400 MHz; JEOL Ltd., Tokyo, Japan) and HRMS on a QExactive Plus (Thermo Fisher Scientific, San Jose, CA, USA) in positive ionization mode coupled to an Acquity UPLC system (Waters Corp., Milford, MA, USA); literature values were compared for structural confirmation (Grove et al., 1952; Levine and Hicks, 1971; Simpson and Holker, 1977). The ^1^H and ^13^C NMR data are included to update the literature (**Supplementary Figure S1**; **Supplementary Table S1**), and this material was used as a standard for the mapping experiments.

### LC-MS Methodology

The QExactive Plus mass spectrometer was scanned across a range from *m/z* 225 to 2000 at a resolution of 70,000. The voltage for both positive and negative ionization modes were set to 3.7 kV, with a nitrogen sheath gas set to 25 arb, and an auxiliary gas set to 5 arb. The S–Lens RF level was set to 50.0 with a capillary temperature at 350°C. The flow rate of the UPLC was set to 0.3 ml/min using a BEH C18 (2.1 mm × 50 mm × 1.7 μm) equilibrated at 40°C. The mobile phase consisted of Fisher Optima LC–MS grade CH_3_CN–H_2_O (acidified with 0.1% formic acid), starting at 15% CH_3_CN and increasing linearly to 100% CH_3_CN over 8 min. It was held at 100% CH_3_CN for 1.5 min before returning to starting conditions for re-equilibration. The PDA was set to acquire from 200 to 500 nm with 4 nm resolution.

### Solid Media Co-cultivation

Fungal cultures of *X. cubensis* (G536) and *P. restrictum* (G85) were transferred separately from PDA solid media with antibiotics onto three plates of MEA separately to act as controls. Simultaneously, six plates of MEA were prepared for co-cultivation of *X. cubensis* (G536) and *P. restrictum* (G85). *X. cubensis* was transferred first and allowed to grow for 10 days because *P. restrictum* grows relatively fast compared to *X. cubensis.* After initiating the co-culture experiments, the plates were then parafilmed and allowed to grow for 30 days, until the cultures began to grow toward each other. Concomitantly, the same experiment was performed using PDA and SDA (Sabouraud Dextrose). Visually, the zone of inhibition was most notable in the MEA plates (data not shown). Thus, the co-cultures grown on MEA were subsequently sampled by droplet–LMJ–SSP.

### Surface Sampling and Mapping

The dropletProbe Premium software converted a CTC/LEAP HTC PAL auto–sampler (LEAP Technologies Inc.) into a droplet–liquid microjunction–surface sampling probe (droplet–LMJ–SSP) (Kertesz and Van Berkel, 2010, 2013; Sica et al., 2015). This probe performs 5 μl microextractions using Fisher Optima LC–MS grade MeOH–H_2_O (1:1). Droplets were dispensed onto the surface of the fungal cultures at a rate of 2 μl/s, held on the surface for 2 s, and withdrawn back into the syringe at the same rate. This extraction process was performed in triplicate using the same droplet. The droplet was then injected into the UPLC-MS system. The relative concentrations of the metabolites, calculated using the dropletProbe Premium software via the area of the exact mass chromatograms for each metabolite at their specific retention times, were mapped, resulting in a visualization of their spatial distribution. The heat mapping experiments focused on griseofulvin (*m/z* 353.0792 ± 5 ppm), which eluted at a retention time of 4.49 ± 0.05 min when using the droplet–LMJ–SSP–UPLC–MS system.

### Stroma Sectioning

**FIGURE 2:**
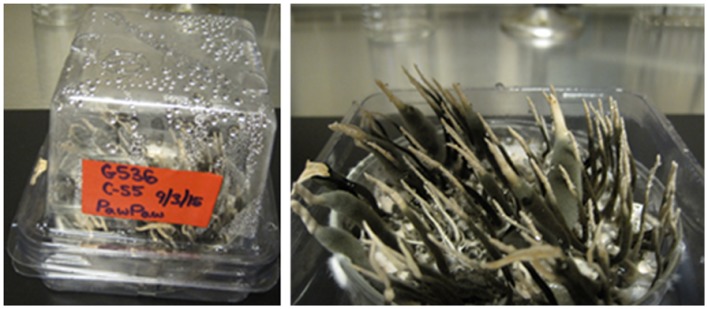
**Images of G536 grown in a glass Petri dish and placed in a sterile plant tissue-cultivating container (Plant Con^®^) to maintain sterile conditions while providing room for stroma growth**.

The fungal culture G536 was inoculated in 10 ml liquid YESD media for 7 days then poured into an autoclaved Petri dish containing autoclaved rice. The Petri dish was then placed inside a sterile plant tissue-cultivating container (Plant Con^®^), and sealed with parafilm. The plant container provides a sterile environment for the stroma producing fungus to grow on rice-based media. Stroma began appearing after 2–3 weeks, but the cultures were allowed to grow for 5.5 weeks (**Figure 2**).

**FIGURE 3:**
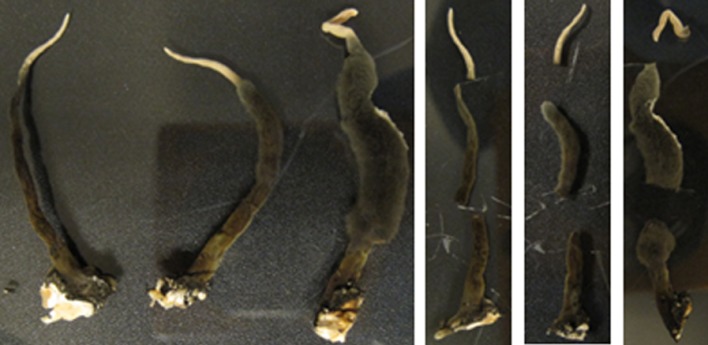
**A representative group (Group 3) of three stroma and their respective segments.** Each segment ranged from 9 to 20 mm.

Stroma were then cut and removed from the Petri dish using a sterile scalpel and forceps. Three individual stroma (one thin [(∼40 mm × 2 mm), one medium (∼50 mm × 4 mm), and one thick (∼50 mm × 6 mm)] were removed from the fungal culture G536. Each stroma was cut into three segments: tip, middle, and base (**Figure 3**; **Supplementary Figure S2**). The three white tips, the three mid-sections, and the three bases were combined and placed into separate vials and weighed. This procedure was repeated two more times resulting in three groups that sampled a total of nine stromata (**Supplementary Table S2**).

To each vial, 5 ml of MeOH–H_2_O (1:1) were added and shaken overnight (∼16 h). The supernatant was then drawn off, placed in a weighed vial and dried under a stream of nitrogen. An aliquot of each dried extract was prepared in MeOH–H_2_O (1:1) to a concentration of 2 mg/ml and subjected to LC-MS analysis.

## Results

### Molecular Analysis

Stromata were examined for sporulating structures, but we were unable to observe any ascospores, asci or conidia. Thus, species delimitation based on morphological characters in cultures of endophytic *Xylaria* was difficult because of a lack of diagnostic characters; therefore, molecular data were used for species identification.

Based on RAxML analysis using *RPB2* sequences (**Supplementary Figure S3**), the strain G536 was identified as *X. cubensis. RPB2* sequences of strain G536 were nested within the *X. cubensis* aggregate (Hsieh et al., 2010) with 80% RAxML bootstrap support. *X. cubensis* (G536) is sister to GQ848365, a *X. cubensis* isolate collected from the Great Smoky Mountains National Park, and forms a monophyletic clade with 100% RAxML bootstrap support with other collections of *X. cubensis* from different geographical locations, including Russia, French West Indies, and Papua New Guinea (**Supplementary Figure S3**). The phylogenetic tree obtained using ITS sequences supported the *RPB2* results. Strain G536 was nested in a clade containing other identified *X. cubensis* sequences, including authenticate voucher sequences of *X. cubensis* (GU373810 and GU991523; **Supplementary Figure S4**). The sequence data were deposited in the GenBank (KU560914, KU560915, KU560916).

### Spatial Distribution of Griseofulvin on *X. cubensis*

**FIGURE 4:**
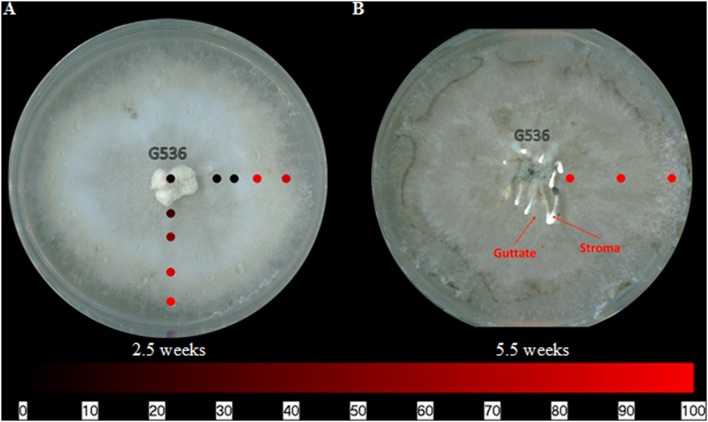
**The spatial distribution of griseofulvin on *X. cubensis* (G536) grown on MEA at **(A)** 2.5 weeks and **(B)** 5.5 weeks of growth displaying the locations of guttate and stroma formations.** Each circle represents a sampled location. Griseofulvin was most intense where the circles are bright red. The bottom bar indicates the relative amount of signal as measured via mass spectrometry.

The griseofulvin-producing fungus, *X. cubensis*, was subjected to surface sampling analysis via the droplet–LMJ–SSP, and the spatial distribution of griseofulvin was mapped. *X. cubensis* was grown on MEA for 2.5 weeks. The culture was then sampled at nine locations, transecting the culture both horizontally and vertically (**Figure 4A**). The first sampling was taken at the point of inoculation (center), followed by four spots toward the right end of the culture and another four spots toward the bottom of the culture (**Figure 4A**). The map shows a higher concentration of griseofulvin toward the edges of the culture. A second culture was simultaneously inoculated and grown for 5.5 weeks. Similarly, this culture was sampled from the center to the edge. However, griseofulvin displayed a more even distribution across the culture (**Figure 4B**). Furthermore, liquid droplets containing high concentrations of secondary metabolites, termed guttates, were formed on the surface of the 5.5 weeks old culture (**Figure 4B**; Figueroa et al., 2014; Singh, 2014).

### Spatial Distribution of Griseofulvin in Stroma of *X. cubensis*

Xylariaceous fungi often grow morphologically distinct ‘finger-like’ protrusions called stroma (**Figure 4B**). Mapping of the spatial distribution of griseofulvin on stroma was originally planned via the droplet–LMJ–SSP, but black, mycelium-covered portions of the stroma were too absorbent to recover the micro-extraction droplet. This experiment was repeated several times to no avail, likely due to the absence of hydrophobins, since aerial mycelium that lack conidia/spores are more hydrophilic (Bayry et al., 2012; Sica et al., 2015). Therefore, an alternative methodology was performed. *X. cubensis* was grown in a sterile plastic container for 5.5 weeks to promote stroma growth. The stroma were then segmented, grouped (**Supplementary Figure S2**), extracted, and then subjected to LC-MS analysis. Griseofulvin was most abundant at the base of the stroma and less abundant as sampled vertically toward the white tip (**Supplementary Figure S5**; **Supplementary Table S2**). For groups 1 and 2, the concentration of griseofulvin in the middle was less than a magnitude greater than the tip, while the base was over three magnitudes greater than the tip. Group 3 varied from this trend with the middle being about three magnitudes greater than the tip, yet the base was only about two magnitudes greater. This discrepancy for group 3 was likely due to the uncharacteristically large mid-section for the thick stroma. Regardless, all three groups displayed increased griseofulvin in the black, mycelium-covered portion of the stroma compared to the white, spore-covered tip.

### Spatial Distribution of Griseofulvin in Co-culturing Experiments

A benefit of mass spectrometry heat mapping experiments of secondary metabolites is not only the ability to provide visualization of the chemical entities, but also, to gain insight into the role that the entities play when interacting with the environment. Mapping the spatial distribution of griseofulvin in a co-culture experiment provides an understanding of the chemical ecology that takes place when the fungus interacts with another fungus. To achieve this, the griseofulvin producing culture, *X. cubensis*, was simultaneously inoculated with a culture of *P. restrictum*, which was chosen due to our knowledge of its chemistry via previous research (Figueroa et al., 2014; Daly et al., 2015).

**FIGURE 5:**
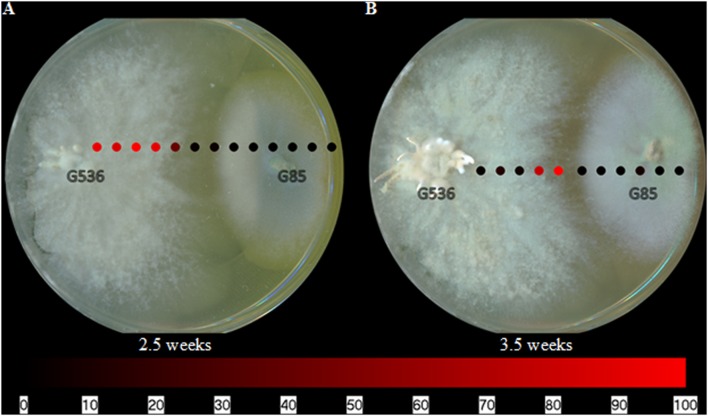
**The spatial distribution of griseofulvin from *X. cubensis* (G536) while grown in co-culture with *P. restrictum* (G85) at **(A)** 2.5 weeks and **(B)** 3.5 weeks.** Each circle represents a sampled location. Griseofulvin was most intense where the circles are bright red. The bottom bar indicates the relative amount of signal as measured via mass spectrometry.

When mapping griseofulvin in the co-culturing experiments, griseofulvin was only detected on the surface of the fungus in which it was produced; it was neither in the surrounding agar nor on the other fungus, *P. restrictum* (**Figure 5**). This distribution was evident after 2.5 and 3.5 weeks of co-cultured fungal growth. While there was a clear inhibition zone between the two fungi, griseofulvin was not detected in this area (**Figure 5**). It remained on the surface of *X. cubensis*, similar to its spatial distribution when grown in isolation (**Figure 4**). Furthermore, *X. cubensis* did not display any physical changes to its growth patterns, besides the inhibition zone.

### The Co-culturing Effects on the Competing *P. restrictum* Culture

**FIGURE 6:**
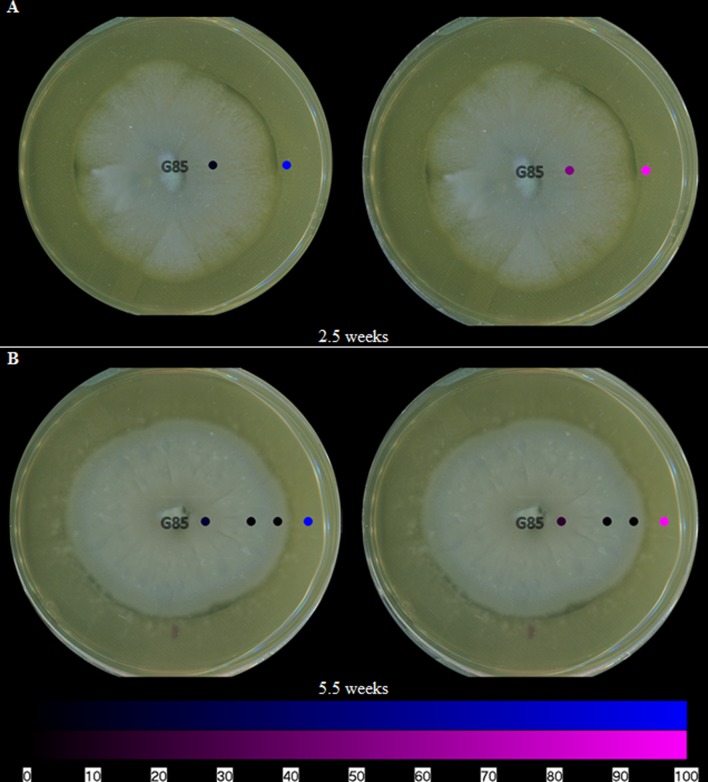
**The spatial distribution of both groups of polyhydroxyanthraquinones on fungal isolates of *P. restrictum* (G85) at **(A)** 2.5 weeks and **(B)** 5.5 weeks.** The color coding (blue and purple) corresponds to the structures in **Figure 1**. Each circle represents a sampled location. The polyhydroxyanthraquinones were most intense where the blue or purple circles were brightest. The bottom bar indicates the relative amount of signal as measured via mass spectrometry.

In addition to mapping the spatial distribution of griseofulvin, it is important to understand the toll that is taken on the other fungal culture. By mapping the metabolites of *P. restrictum*, the inhibitory effects of griseofulvin can be further understood. In a previous study of *P. restrictum*, a series of polyhydroxyanthraquinones were identified (Figueroa et al., 2014; Daly et al., 2015). In this study, *P. restrictum* was grown on agar for 2.5 weeks, and five of its secondary metabolites (compounds 2–6) (**Figure 1**) were readily detected on the surface using the droplet–LMJ–SSP (**Figure 6A**). While the metabolites were detected on the surface of the mycelium, their signals were a magnitude greater on the surface of the agar (**Supplementary Figure S6**). Furthermore, another plate of this culture was grown for 5.5 weeks, and similar distributions (**Figure 6B**) and magnitude differences (**Supplementary Figure S7**) were observed.

**FIGURE 7:**
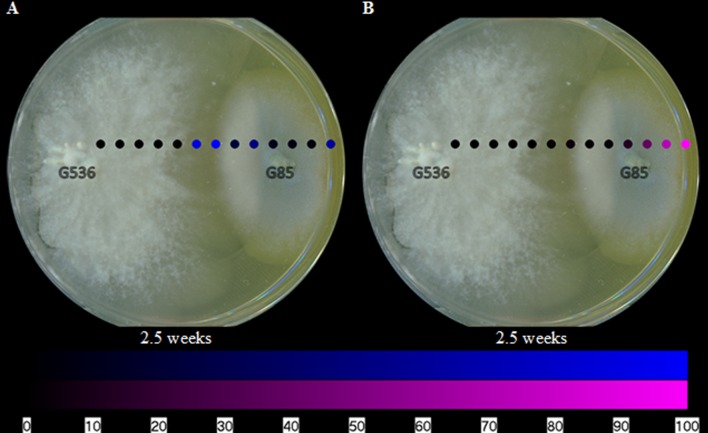
**The spatial distribution of the **(A)** blue and **(B)** purple groups of polyhydroxyanthraquinones on fungal isolates of *P. restrictum* (G85) while grown in co-culture with *X. cubensis* (G536) at 2.5 weeks.** The color coding (blue and purple) corresponds to the structures in **Figure 1**. Each circle represents a sampled location. The polyhydroxyanthraquinones were most intense where the blue or purple circles were brightest. The bottom bar indicates the relative amount of signal as measured via mass spectrometry.

Visualizing the secondary metabolites from *P. restrictum* in isolation created a baseline of how this fungal culture distributes its metabolites. Subsequently, by mapping the metabolites of *P. restrictum* in co-culture with *X. cubensis*, the effects of griseofulvin on a competing fungus could be probed. The five compounds from *P. restrictum* were grouped into two categories (compounds 2–3; purple and compounds 4–6; blue; **Figure 1**) that were determined by their distribution patterns in co-culture (**Figure 7**). As a fungal isolate, *P. restrictum* metabolites (2–6) were exuded into the surrounding agar (**Figure 6**); however, in co-culture with *X. cubensis*, only three of the five compounds (4–6; blue) continued this trend by being detected on both sides of the colony (**Figure 7A**). The other two metabolites (2–3; purple) were not detected in the interaction zone, but still remain exuded into the media on the side of the colony farthest from the griseofulvin-producing fungus (**Figure 7B**).

**FIGURE 8:**
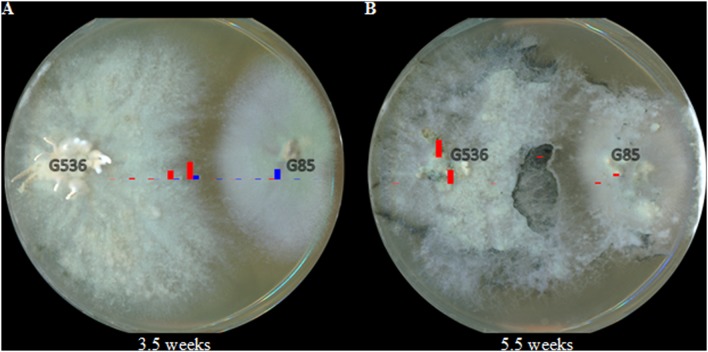
**Heat map of griseofulvin (red) and the *P. restrictum* (G85) metabolites (blue group only; purple metabolites were undetectable) at **(A)** 3.5 weeks and **(B)** 5.5 weeks.** The heights of the bars are relative to their intensity from the HRMS data.

As the co-cultures continued to grow for another week to a total of 3.5 weeks, the pattern continued (**Figure 8A**). Griseofulvin still remained primarily on *X. cubensis*, while some of the *P. restrictum* (G85) metabolites were no longer detectable (2–3; purple). By 5.5 weeks, griseofulvin was detected on both the original *X. cubensis* colony and where the original *P. restrictum* colony was inoculated (**Figure 8B**). This was attributed to the observation that new growths of *X. cubensis* had begun to grow where the original *P. restrictum* colony was, as indicated by the formation of guttates and stroma containing griseofulvin. This suggests how griseofulvin may be used by *X. cubensis* to outcompete other fungi and then propagate.

**FIGURE 9:**
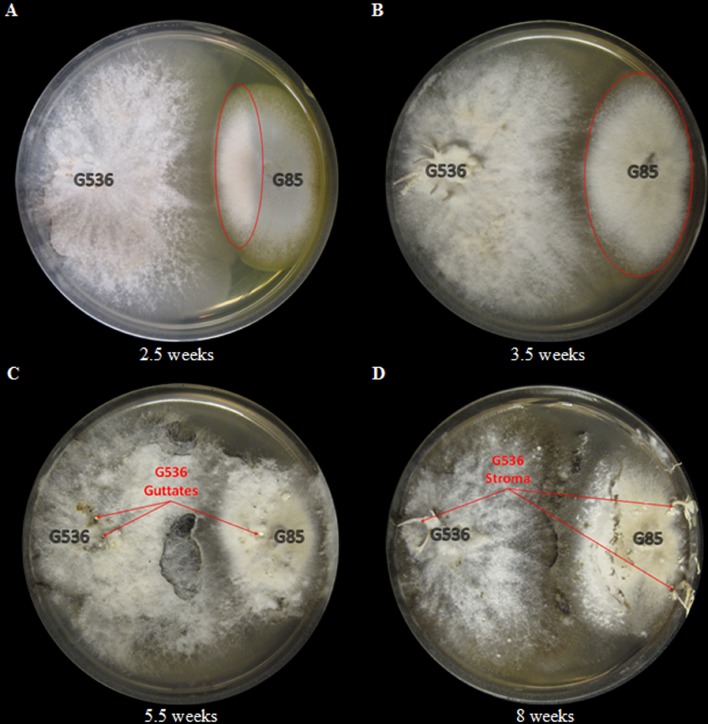
**The visible discoloration of *P. restrictum* (G85) while in co-culture with the griseofulvin-producer, *X. cubensis* (G536), at **(A)** 2.5 weeks and **(B)** 3.5 weeks.** The discolored regions of *P. restrictum* (G85) are circled in red. The visible expansion of *X. cubensis* (G536) while in co-culture with *P. restrictum* (G85) at **(C)** 5.5 weeks and **(D)** 8 weeks. The guttates were attributed to *X. cubensis* (G536) due to the detection of griseofulvin. The stroma were attributed to *X. cubensis* (G536) since *P. restrictum* (G85) does not produce stroma.

Visually, the growth of the competing culture, *P. restrictum*, began to lose color after 2.5 weeks. (**Figure 9A**). The left most edge of *P. restrictum* began to turn white, something that was not observed when this fungus was grown in isolation. The discoloration continued to grow, eventually turning the entire *P. restrictum* colony white after 3.5 weeks of co-culturing (**Figure 9B**). After 5.5 weeks, the *P. restrictum* culture failed to grow further and the griseofulvin-producer, *X. cubensis*, began to grow new colonies on top of *P. restrictum* (**Figure 9C**). Guttates formed on both the original and the new growths of *X. cubensis*. Furthermore, the formation of stroma, morphological characteristics of fungi of this family, were visible on both colonies as well. By 8 weeks, the stromata from *X. cubensis* were prevalent on both the original colony and the new growths that were on top of the presumed dead *P. restrictum* colony (**Figure 9D**).

## Discussion

### Spatial Distribution of Griseofulvin on *X. cubensis*

The heat mapping experiments of the griseofulvin-producer, *X. cubensis* (G536), showed a relative concentration of griseofulvin toward the edges after 2.5 weeks of growth (**Figure 4A**). As the culture grew for 5.5 weeks, the distribution appeared more uniform across the culture (**Figure 4B**). It is hypothesized that griseofulvin is most abundant in the younger sections of a colony (i.e., the outer edges) to facilitate continued growth, but evens out when the culture is no longer growing laterally.

**FIGURE 10:**
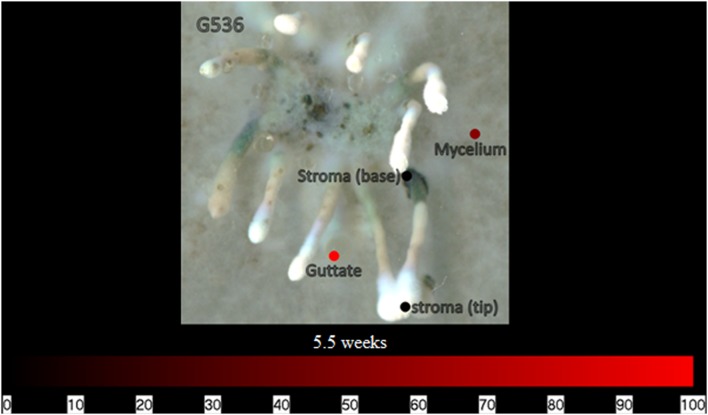
**The spatial distribution indicating the relative intensities of griseofulvin on fungal culture *X. cubensis* (G536) for the stroma, mycelium, and guttates at 5.5 weeks.** Each circle represents a sampled location. Griseofulvin was most intense where the circles are bright red. The bottom bar indicates the relative amount of signal as measured via mass spectrometry.

Additionally, at 3.5 weeks, the culture began to form stromata and produce guttates (i.e., liquid droplets; Figueroa et al., 2014; Singh, 2014) on its surface. On a culture at 5.5 weeks, surface sampling analysis was performed using the droplet–LMJ–SSP to analyze the stromata and guttates. The signal of griseofulvin on a guttate was over two magnitudes greater than that of a stroma (both base and tip) and about half a magnitude greater than on the mycelium (**Figure 10**; **Supplementary Figure S8**). This finding continues to support a previous hypothesis that guttates are concentrated droplets of metabolites exuded into their environment (Wang et al., 2010; Sica et al., 2015; Paguigan et al., 2016). In this culture, the stroma were immature, thus the black, mycelium-covered body was not as hydrophilic as it was for the older stroma. As such, the droplet–LMJ–SSP was able to carry out the analysis *in situ*.

### Spatial Distribution of Griseofulvin in Stroma of *X. cubensis*

Initially, the stroma were to be analyzed via the droplet–LMJ–SSP to give a heat map from the base of a stroma to the tip. However, upon sampling the black, mycelium-covered portions of the stroma, the droplet was quickly absorbed in the stroma, rendering the droplet unrecoverable. The white tips of the stroma were amenable to droplet recovery, but not the black regions of the stroma, which were more heavily covered by mycelium. Various methods were explored in an effort to directly sample the stroma using the droplet–LMJ–SSP but all proved unsuccessful. Three of these stroma modifications included: (1) freezing stroma in liquid nitrogen, (2) drying stroma in a desiccator, or (3) cutting stroma open longitudinally. None of these methods worked and all resulted in the droplet being absorbed into the stroma. Combinations of all of these methods were also attempted with no success. This led to the more traditional procedure of cutting the stroma into three segments, extracting, and then analyzing via LC-MS. The results indicated that griseofulvin was most abundant at the base compared to the tip. Since griseofulvin was concentrated toward the edges of the mycelial growth (**Figure 4A**), compounded with the observation that griseofulvin was concentrated toward the base of the stroma, we hypothesized that the stroma were basipetal (i.e., youngest at the base of the stromata).

### Spatial Distribution of Griseofulvin in Co-culturing Experiments

Since griseofulvin is known to be fungistatic and not fungicidal, we hypothesized that griseofulvin would reside mostly on the surface of *X. cubensis*, rather than actively being exuded into its surroundings. This would inhibit the growth of other fungi, rather than kill them. This contrasts a phenomenon that was previously reported when mapping the spatial distribution of the fungicidal compound, iturin (Klich et al., 1994), from a coral microbe (Moree et al., 2013) in co-culture with a fungus. In that case, iturin was exuded into the media, thus similarly inhibiting the fungal growth and the production of fungal secondary metabolites (Moree et al., 2013).

The spatial distribution patterns of griseofulvin as an isolated fungal culture (**Figure 4**) and as part of a co-culture experiment (**Figure 5**) displayed minimal changes. Griseofulvin appeared to remain on the surface of *X. cubensis*, rather than exuding into the surrounding media or on the competing fungus. These mapping experiments support the fungistatic ability of griseofulvin by remaining on the griseofulvin-producing fungus.

The fungistatic ability of griseofulvin was explored further by growing *X. cubensis* along with another fungus. The fungus selected was a non-xylariaceous endophyte from *S. marianum* (milk thistle) and was previously identified as *P. restrictum*, coded G85 (Figueroa et al., 2014). *P. restrictum* was selected for three reasons: (1) the secondary metabolites of this fungal culture were defined (Figueroa et al., 2014); (2) the key secondary metabolites were explored previously using mass spectrometry imaging experiments (Figueroa et al., 2014); (3) the secondary metabolites were shown to play a role in inhibiting the quorum sensing pathways for bacteria (Daly et al., 2015), hence we suspected that they would also be found exuded into the environment.

### The Co-culturing Effects on the Competing *P. restrictum* Culture

The spatial distribution of the metabolites from fungal culture G85 were mapped using the droplet–LMJ–SSP. The metabolites were exuded into the surrounding agar of *P. restrictum* (**Figure 6**). This behavior was not unexpected since we presumed that these compounds would be exuded into the environment due to our understanding of their biological activity as microbial quorum quenching metabolites (Figueroa et al., 2014; Daly et al., 2015). However, while in co-culture with *X. cubensis*, some of the *P. restrictum* metabolites (purple) were not observed in the interaction zone between fungal cultures *P. restrictum* and *X. cubensis* (**Figures 7** and **8**), indicating inhibition of secondary metabolite biosynthesis in *P. restrictum*. Interestingly, the two compounds (2–3; purple) most affected by the co-culturing experiment were the same two compounds that were most active in the bacterial quorum sensing inhibition bioassay (Figueroa et al., 2014; Daly et al., 2015).

From an ecological standpoint, the growth of the competing culture, *P. restrictum*, was clearly inhibited as observed by its loss of color over time. Ultimately, this discoloration was attributed to *P. restrictum* dying, since the culture never continued to grow in the direction where the white was present. However, griseofulvin is reported to be fungistatic rather than fungicidal (Robinson, 1960; Corvis et al., 2006), therefore, it was questioned whether or not *X. cubensis* truly killed *P. restrictum* prior to growing over it. An experiment, coined the Lazarus experiment (John 11:1–44 (Common English Bible)), was performed to test if *P. restrictum* survived the co-culture experiment with *X. cubensis*. Therefore, *P. restrictum* was subsequently transferred from the co-culture Petri dish to a new Petri dish without any competing fungal cultures. Within a week, *P. restrictum* began to grow a new colony, thus confirming that it wasn’t killed by the griseofulvin from *X. cubensis*, supporting the fungistatic properties of griseofulvin.

### *Xylaria cubensis* Lives a Dual Mode of Life

*Xylaria cubensis* is widely distributed in tropical, subtropical, and temperate regions of the world, where it is usually found in decaying angiosperm wood (Rogers, 1984; Whalley et al., 2015). Additionally, there is growing evidence in the literature where *X. cubensis* has been reported as an endophyte from various healthy tissue types (twigs and foliage; Rodrigues, 1994; Frohlich et al., 2000; Davis et al., 2003; Okane et al., 2008, 2012). The production of griseofulvin by the endophytic stage of *X. cubensis* has an ecological advantage to the fungus. Similarly, other studies of endophytic *Xylaria* have also reported griseofulvin as a major secondary metabolite (Park et al., 2005). By producing griseofulvin, *X. cubensis* can stunt the growth of other fungi in the host in an endophytic state or on decaying substrates in the saprobic state. The production of secondary compounds is a common characteristic of many endophytic fungi and provides a basis for selection supporting the symbiosis in the host plant (Carroll, 1988). Thus, by inhibiting the growth of other fungi, *X. cubensis* ensures it can spread its mycelium throughout the host when its host senesces, at which time it can begin to sustain as a saprobe by decomposing cell wall materials (Petrini et al., 1995; Whalley, 1996); griseofulvin biosynthesis likely imparts a competitive advantage in the saprobic state as well.

Mass spectrometry mapping experiments enabled the visualization of how griseofulvin biosynthesis imparts an ecological advantage to a fungus, which can lead dual (endophytic/saprobic) modes of life. The measurements of the spatial and temporal production of griseofulvin by endophytic *Xylaria* sp. when in competition with another endophyte demonstrated how endophytes might use secondary metabolites against other microorganisms in nature. Our chemistry data lend support to the hypothesis that *Xylaria* endophytes are quiet colonizers of their host. Presumably, they use secondary metabolites, such as griseofulvin, to keep other microbes in check. This allows them to spread within the host, so it can decompose the plant when it begins to senesce (Petrini et al., 1995; Whalley, 1996).

## Conclusion

Ambient mass spectrometry mapping techniques provided an understanding of the chemical ecology that took place between two fungal cultures. For *X. cubensis*, it was revealed that griseofulvin was concentrated in the younger tissues of the fungus, typically around 2–3 weeks of development, toward the edges of the mycelial growth and at the base of the stroma. Conversely, *P. restrictum* excreted the secondary metabolites, polyhydroxyanthraquinones, into the surrounding environment with only trace amounts detected on the mycelium. When *X. cubensis* was grown in co-culture with *P. restrictum*, the spatial and temporal distributions of griseofulvin remained the same, but there was a noticeable effect on the distribution of polyhydroxyanthraquinones from *P. restrictum*. The biosyntheses of two of the five polyhydroxyanthraquinones were greatly hindered. Eventually, the growth of *P. restrictum* was inhibited and *X. cubensis* began to overtake the culture. Griseofulvin displayed clear signs of having a fungistatic effect on competing fungi as visualized via mass spectrometry mapping experiments. By exploring the temporal and spatial distributions of fungal secondary metabolites through these co-culturing experiments, the questions of – *where* (spatial), *when* (temporal), *what* (qualitative), *how much* (quantitative), *why* (function), and *which* (target) – were probed in detail.

## Author Contributions

VS carried out the mass spectrometry mapping experiments. He also helped with the general design of the experiments and the writing of the manuscript. HR dealt with the mycology aspects of the science, including molecular identification of fungi and growth of the fungi. He also assisted with the writing of the manuscript. ER isolated griseofulvin under the direction of VS. ET assisted with the isolation of the fungal endophyte from the plant. RB prepared the stroma for measurements and maintained cultures for the co-culture experiments. NO led the project, particularly the experimental design, interpretation of data, and writing of manuscript.

## Conflict of Interest Statement

The authors declare that the research was conducted in the absence of any commercial or financial relationships that could be construed as a potential conflict of interest.
