# Supplementary material for: Spatial and Temporal Profiling of Griseofulvin Production in Xylaria cubensis Using Mass Spectrometry Mapping
Source: Front Microbiol. 2016 Apr 26;7:544. doi: 10.3389/fmicb.2016.00544 (PMC4844619; doi:10.3389/fmicb.2016.00544)
Supplement: Supplementary file 1 [file Data_Sheet_1.DOCX]

**Spatial and temporal profiling of griseofulvin production in *Xylaria cubensis* using mass spectrometry mapping**

Vincent P. Sica, Evan R. Rees, Edem Tchegnon, Robert H. Bardsley IV, Huzefa A. Raja, and Nicholas H. Oberlies*

**SUPPORTING INFORMATION**

**Figure S1.** ^1^H (400 MHz; top) and ^13^C NMR (100 MHz; middle) NMR data (both acquired in CDCl_3_) and the HRMS data for griseofulvin. The HRMS data was 0.0 ppm for C_17_H_18_O_6_Cl ([M+H]^+^ from calculated (*m/z* 353.0792).

**Figure S2.** The three groups of stroma (1, 2, 3) and the length of their respective segments (A, B, C) in mm.

**Figure S3.** Phylogram of the most likely tree (-lnL = 29759.75) from a RAxML analysis of 95 sequences based on partial RPB2 data (1236 bp). Numbers refer to RAxML bootstrap support values ≥ 70% based on 1000 replicates. Bar indicates nucleotide substitutions per site. Strain G536 is identified as *Xylaria cubensis*, (bold, and highlighted in gray).

**Figure S4.** Phylogram of the most likely tree (-lnL = 1351.36) from a RAxML analysis of 35 sequences based on ITS data (568 bp). Numbers refer to RAxML bootstrap support values ≥ 50% based on 1000 replicates. Bar indicates nucleotide substitutions per site. Strain G536 is identified as *Xylaria cubensis* (bold) as it is nested with an authenticate voucher collection (JDR 860, GU991523).

**Figure S5.** The base peak chromatograms for the stromata of *X. cubensis* (G536) displayed a significant increase in detection of griseofulvin (boxed in red) in the base.

**Figure S6.** Secondary metabolites (**2**-**6**) of *P. restrictum* after 2.5 weeks on the (**A**) mycelium and (**B**) agar.

**Figure S7.** Secondary metabolites (**2**-**6**) of *P. restrictum* after 5.5 weeks on the (**A**) mycelium and (**B**) agar.

**Figure S8.** Detection of griseofulvin (**1**) on the surface of a guttate, mycelium, and stromata (base and tip) for *X. cubensis*.

**Table S1.** ^1^H (400 MHz) and ^13^C NMR (100 MHz) data for griseofulvin in CDCl_3_.

**Table S2.** The lengths and weights for the three groups of stroma used for the spatial distribution of griseofulvin along the stroma. Each segment (i.e. Group 1A, Group 1B, etc.) was extracted and subjected to LC-MS. The area under the curve (AUC) for griseofulvin (*m/z* 353.0792 ± 5 ppm) was compared for each segment.

| ****  (**1**) |
| --- |
| **** |
| **** |
| **** |
| **Figure S1.** ^1^H (400 MHz; top) and ^13^C NMR (100 MHz; middle) NMR data (both acquired in CDCl_3_) and the HRMS data for griseofulvin. The HRMS data was 0.0 ppm for C_17_H_18_O_6_Cl ([M+H]^+^ from calculated (*m/z* 353.0792). |

| Group 1 | Group 2 | Group 3 |
| --- | --- | --- |
| 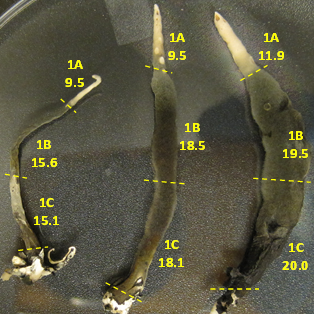 | 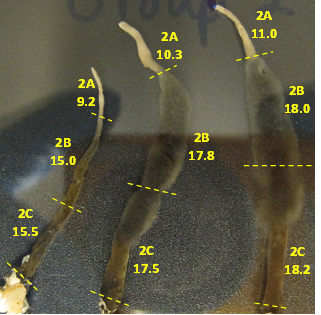 |  |
| **Figure S2.** The three groups of stroma (1, 2, 3) and the length of their respective segments (A, B, C) in mm. | | |

| 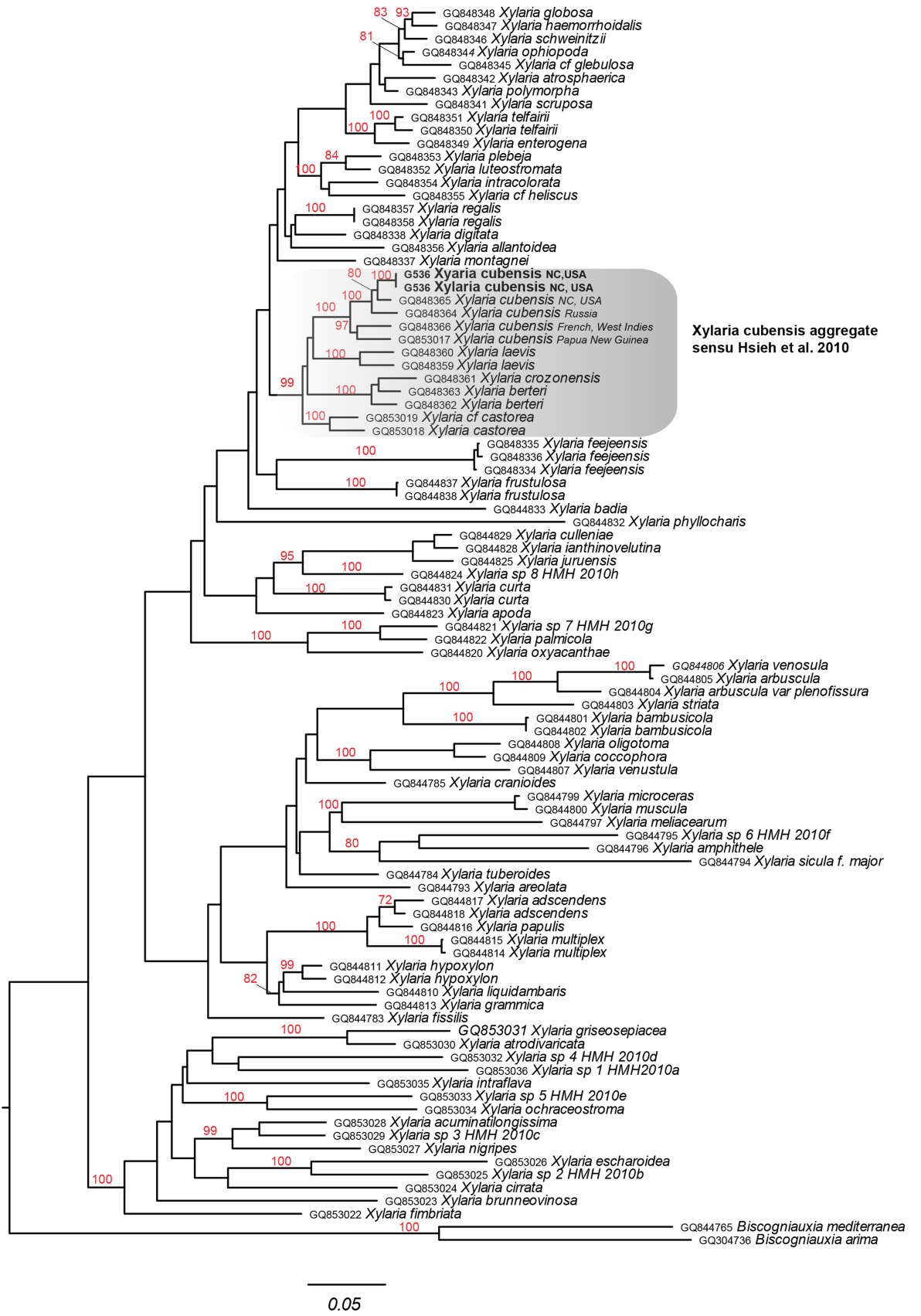 |
| --- |
| **Figure S3.** Phylogram of the most likely tree (-lnL = 29759.75) from a RAxML analysis of 95 sequences based on partial RPB2 data (1236 bp). Numbers refer to RAxML bootstrap support values ≥ 70% based on 1000 replicates. Bar indicates nucleotide substitutions per site. Strain G536 is identified as *Xylaria cubensis*, (bold, and highlighted in gray). |

| 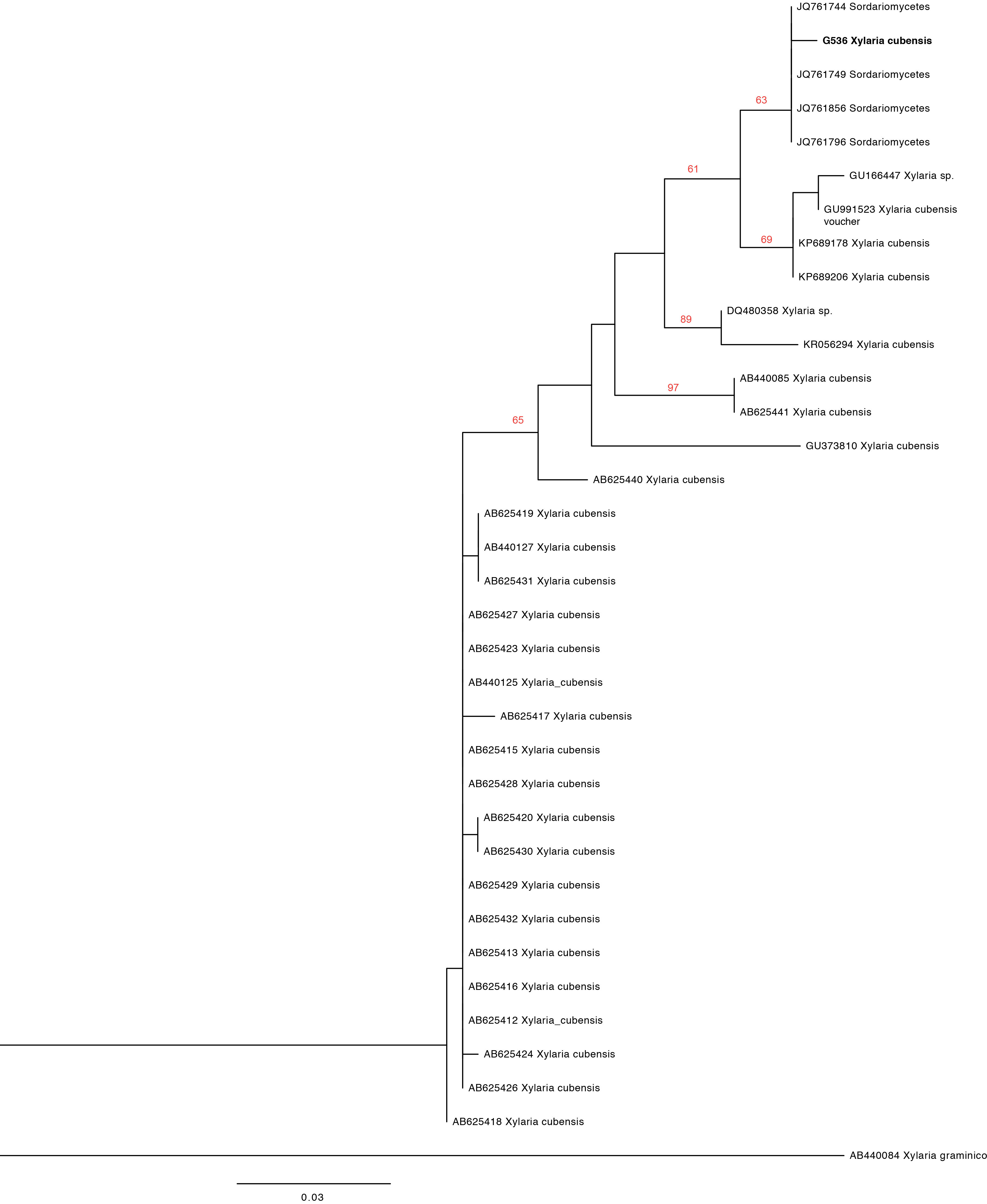 |  |
| --- | --- |
| **Figure S4.** Phylogram of the most likely tree (-lnL = 1351.36) from a RAxML analysis of 35 sequences based on ITS data (568 bp). Numbers refer to RAxML bootstrap support values ≥ 50% based on 1000 replicates. Bar indicates nucleotide substitutions per site. Strain G536 is identified as *Xylaria cubensis* (bold) as it is nested with an authenticate voucher collection (JDR 860, GU991523). |  |
| 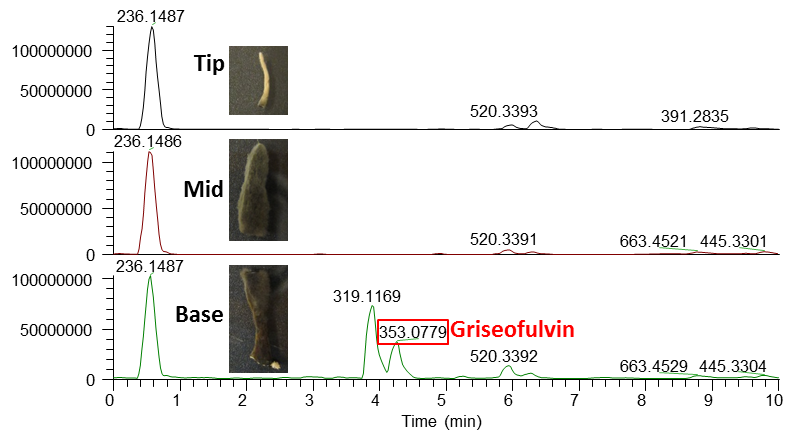 | |
| **Figure S5.** The base peak chromatograms for the stromata of *X. cubensis* (G536) displayed a significant increase in detection of griseofulvin (boxed in red) in the base. | |

| **A** | | Mycelium | 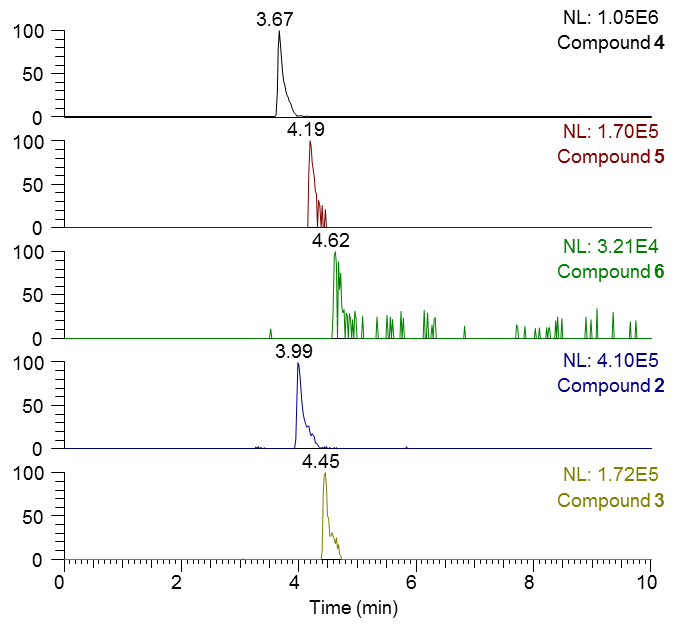 |
| --- | --- | --- | --- |
| **B** | Agar | | 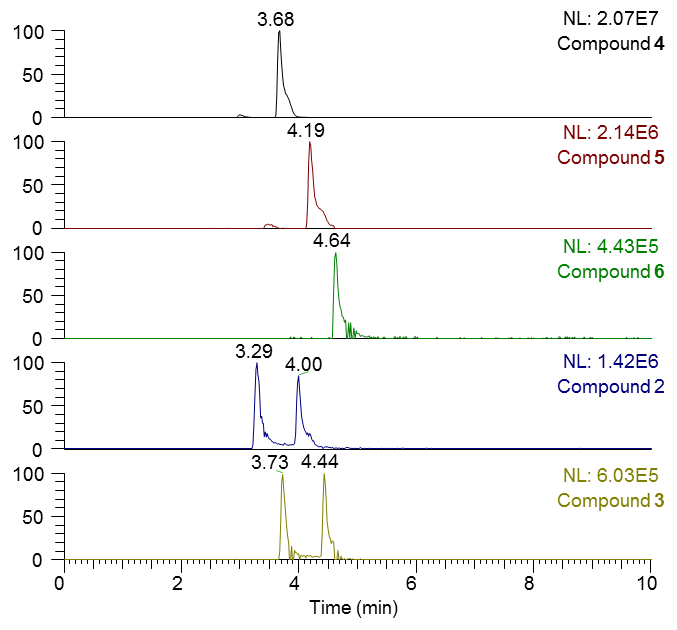 |
| **Figure S6.** Secondary metabolites (**2**-**6**) of *P. restrictum* after 2.5 weeks on the (**A**) mycelium and (**B**) agar. | | | |

| **A** | Mycelium | 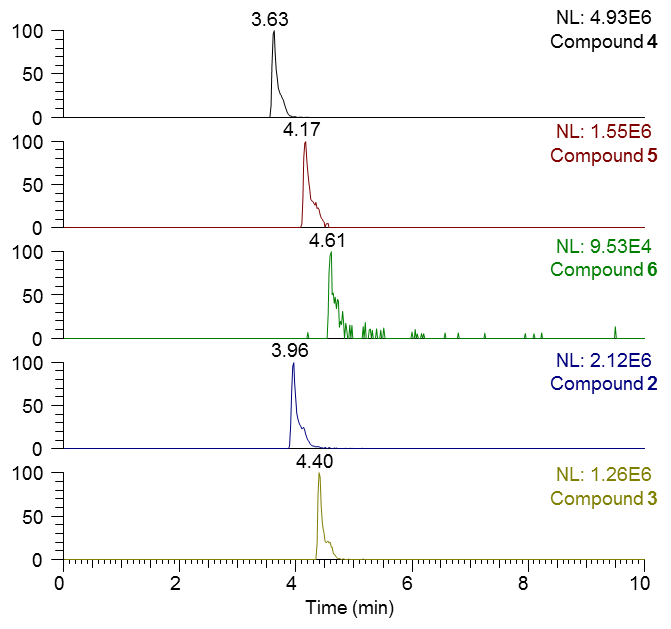 |
| --- | --- | --- |
| **B** | Agar | 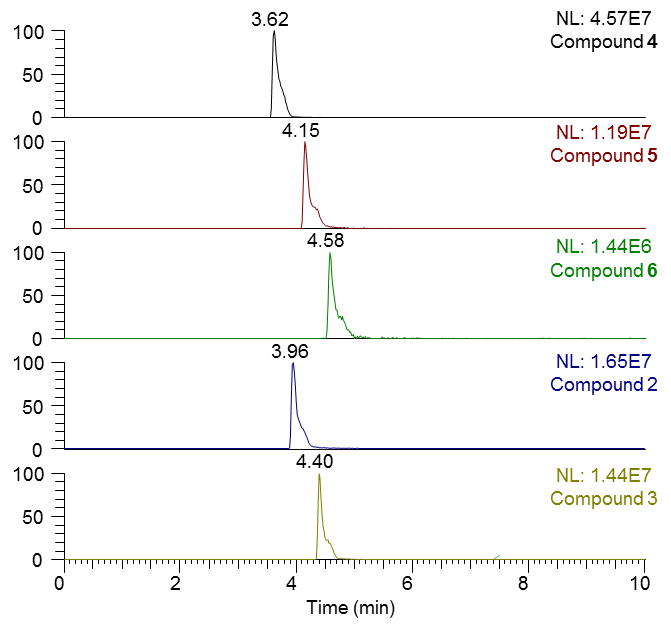 |
| **Figure S7.** Secondary metabolites (**2**-**6**) of *P. restrictum* after 5.5 weeks on the (**A**) mycelium and (**B**) agar. | | |

| **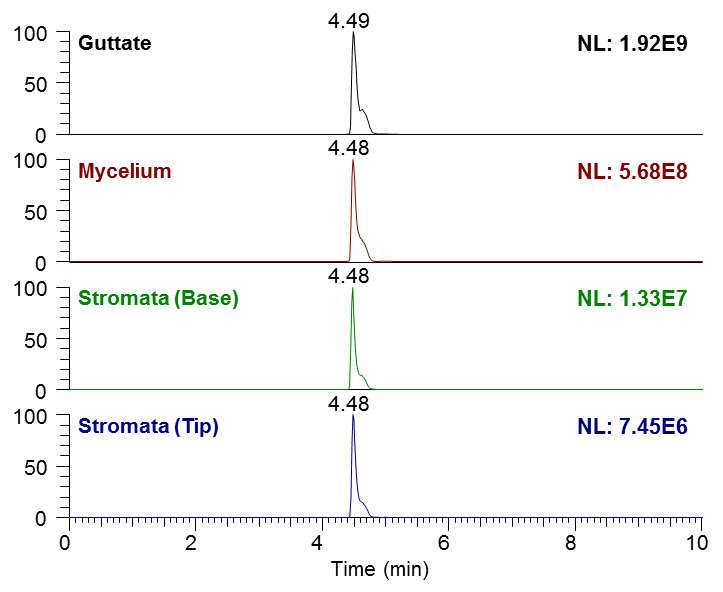** |
| --- |
| **Figure S8.** Detection of griseofulvin (**1**) on the surface of a guttate, mycelium, and stromata (base and tip) for *X. cubensis*. |

| **Table S1.** ^1^H (400 MHz) and ^13^C NMR (100 MHz) data for griseofulvin in CDCl_3_. | | |
| --- | --- | --- |
| **** | | |
| # | δ_H_ (mult., *J*) | δ_C_ |
| 1 | 0.97 (d, 6.75) | 14.3 |
| 2 | 2.85 (m, 4.72, 6.80, 13.66) | 36.4 |
| 3 | 2.45 (dd, 4.64, 16.57)  3.04 (dd, 13.36, 16.57) | 40.1 |
| 4 |  | 197.2 |
| 5 | 5.54 (s) | 104.9 |
| 6 |  | 170.9 |
| 7 |  | 90.8 |
| 8 |  | 192.6 |
| 9 |  | 105.1 |
| 10 |  | 157.8 |
| 11 | 6.13 (s) | 89.5 |
| 12 |  | 169.6 |
| 13 |  | 97.2 |
| 14 |  | 164.7 |
| 6-OMe | 3.62 (s) | 56.8 |
| 10-OMe | 3.98 (s) | 56.5 |
| 12-OMe | 4.03 (s) | 57.1 |

| **Table S2.** The lengths and weights for the three groups of stroma used for the spatial distribution of griseofulvin along the stroma. Each segment (i.e. Group 1A, Group 1B, etc.) was extracted and subjected to LC-MS. The area under the curve (AUC) for griseofulvin (*m/z* 353.0792 ± 5 ppm) was compared for each segment. | | | | | |
| --- | --- | --- | --- | --- | --- |
| Group 1 | | | | | |
|  | Thin (mm) | Medium (mm) | Thick (mm) | Combined weight (mg) | AUC |
| A (top) | 9.5 | 9.5 | 11.9 | 26.79 | 4.84 × 10^4^ |
| B (mid) | 15.6 | 18.5 | 19.5 | 89.18 | 1.92 × 10^5^ |
| C (base) | 15.1 | 18.1 | 20.0 | 99.21 | 1.04 × 10^8^ |
| Group 2 | | | | | |
|  | Thin (mm) | Medium (mm) | Thick (mm) | Combined weight (mg) | AUC |
| A (top) | 9.2 | 10.3 | 11.0 | 6.55 | 7.77 × 10^4^ |
| B (mid) | 15.0 | 17.8 | 18.0 | 42.25 | 6.49 × 10^5^ |
| C (base) | 15.5 | 17.5 | 18.2 | 52.35 | 3.01 × 10^8^ |
| Group 3 | | | | | |
|  | Thin (mm) | Medium (mm) | Thick (mm) | Combined weight (mg) | AUC |
| A (top) | 10.0 | 16.5 | 10.2 | 5.10 | 2.50 × 10^5^ |
| B (mid) | 15.5 | 16.5 | 18.3 | 36.48 | 2.21 × 10^8^ |
| C (base) | 16.0 | 10.7 | 19.0 | 57.70 | 6.53 × 10^7^ |
